# A New Flow-Regulating Cell Type in the Demosponge *Tethya wilhelma* – Functional Cellular Anatomy of a Leuconoid Canal System

**DOI:** 10.1371/journal.pone.0113153

**Published:** 2014-11-19

**Authors:** Jörg U. Hammel, Michael Nickel

**Affiliations:** Institut für Spezielle Zoologie und Evolutionsbiologie mit Phyletischem Museum, Friedrich-Schiller-Universität Jena, Erbertstr. 1, 07743, Jena, Germany; University of Missouri, United States of America

## Abstract

Demosponges possess a leucon-type canal system which is characterized by a highly complex network of canal segments and choanocyte chambers. As sponges are sessile filter feeders, their aquiferous system plays an essential role in various fundamental physiological processes. Due to the morphological and architectural complexity of the canal system and the strong interdependence between flow conditions and anatomy, our understanding of fluid dynamics throughout leuconoid systems is patchy. This paper provides comprehensive morphometric data on the general architecture of the canal system, flow measurements and detailed cellular anatomical information to help fill in the gaps. We focus on the functional cellular anatomy of the aquiferous system and discuss all relevant cell types in the context of hydrodynamic and evolutionary constraints. Our analysis is based on the canal system of the tropical demosponge *Tethya wilhelma*, which we studied using scanning electron microscopy. We found a hitherto undescribed cell type, the reticuloapopylocyte, which is involved in flow regulation in the choanocyte chambers. It has a highly fenestrated, grid-like morphology and covers the apopylar opening. The minute opening of the reticuloapopylocyte occurs in an opened, intermediate and closed state. These states permit a gradual regulation of the total apopylar opening area. In this paper the three states are included in a theoretical study into flow conditions which aims to draw a link between functional cellular anatomy, the hydrodynamic situation and the regular body contractions seen in *T. wilhelma*. This provides a basis for new hypotheses regarding the function of bypass elements and the role of hydrostatic pressure in body contractions. Our study provides insights into the local and global flow conditions in the sponge canal system and thus enhances current understanding of related physiological processes.

## Introduction

Sponges are sessile filter-feeding animals. Accordingly, the canal or aquiferous system is their most distinct anatomical feature. Functionally speaking it can be considered the most important organizational unit besides the skeletal elements which give the sponge its structure. In accordance with their feeding habits, all physiological processes in sponges rely on the ability to process high volumes of water through the body. Only in this way are they able to obtain the required nutrients and oxygen and get rid of metabolic waste products.

**Figure 1:**
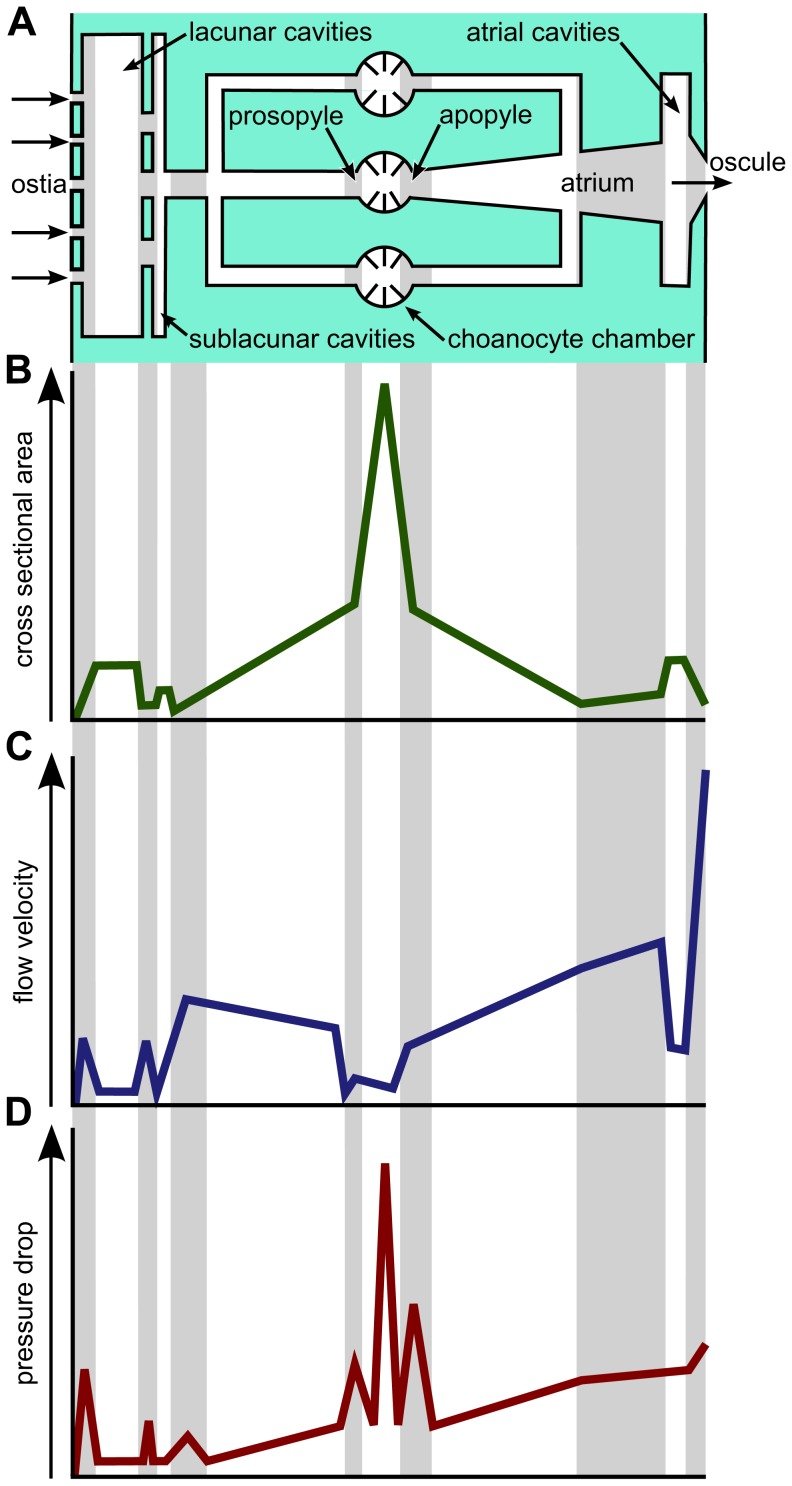
Scheme of hydrodynamic conditions in different sections of the leuconoid canal system based on morphometric and anatomical data on the sponge canal system as well as on fundamental physical laws in hydrodynamics [3], [4], [6], [9]–[11], [46]. (A) Structural representation of the main canal system elements in the direction of flow. (B) Schematic diagram of the change of available total cross sectional area along the flow path. (C) Schematic diagram of flow velocities in the canal system. (D) Schematic diagram of the change of pressure drop along the flow path.

Research into the biomechanics and fluid dynamics of filter-feeding and into biological fluid transport systems in general has revealed a close interdependence between hydrodynamic constraints, the micro- and macro-morphology of the cellular elements involved and, indeed, the structure of the anatomy in its entirety [1]–[6]. A number of hydrodynamic constraints and optimality principles have been suggested to play a role in shaping the general architecture of the canal system [3], but the key features appear to be flow resistance and pressure drop [2]. Pressure drop can be understood as the resistance which fluid encounters when it passes through a filter. In the incurrent canal system in sponges, small apertures in the form of ostia and prosopyles contribute significantly to the pressure drop within the system (Figure 1). Further on, the apopylar apertures and the microvilli collar of the choanocyte chambers are also thought to play a significant role (Figure 1). While the effect of pressure drop in sponges has been considered to varying extents in general models of flow on an organismal scale, almost nothing is known about the influence of cell morphologies on local flow conditions or their implication for hydrodynamics on an organismal scale. Local flow regimes are of the utmost importance, however, especially when it comes to functional considerations such as nutrient uptake and gas exchange.

From a biological perspective resistance has a significant influence on two central aspects of filter feeding. On the one hand it determines the power required to move the fluid through the system. On the other hand it determines, in the context of morphological constraints and anatomy, the flow velocity of the fluid in the canal system. Particle capture rates are greatly influenced by the prevailing Reynolds number and are therefore related to flow velocity and anatomy [7], [8]. We are consequently faced with complex interdependencies between cellular morphology and anatomy, energy expenditure and filter-feeding. In order to understand these complex relationships in sponges we need detailed information regarding the hierarchy and three-dimensional architecture of the canal system, quantitative morphometric data pertaining to individual canal segments, flow velocity measurements and detailed morphological data regarding the cellular entities involved in the canal system. The morphometric and anatomical data pertaining to the architecture of the canal system and the cell types involved then needs to be integrated into basic fluid dynamic theory in order to gain a deeper and more detailed understanding of the hydrodynamic situation as a whole in sponge canal systems. Current understanding is based on general information regarding leucon-type canal systems [3], [4], [9], [10] and recent specific morphometric and hierarchical data pertaining to the aquiferous system [6]. Flow velocity within the canal system is affected most prominently by the total available cross-sectional area of every functional unit in it (Figure 1A–C) [3], [4], [11]. Slower flow velocities are caused by an increase in total available cross-sectional area on any given hierarchical level [4], [9]. However, the cross-sectional area of single segments on a hierarchical level is usually small. Overall increases in cross-sectional area are related to increases in the number of small sized segments on the respective level [6], [12]. As the lower cross-sectional area of small sized canals is a consequence of their smaller diameter we can draw from the following two equations a direct relationship between pressure drop and resistance:
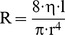
(1)

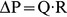
(2)Where R is resistance, η the viscosity of the fluid, l the length of a canal segment, r the canal diameter, ΔP is pressure drop and Q is flow. According to equation (1), radius has the greatest influence on resistance, which allows us to conclude that numerous small sized canals will lead to high resistance and therefore necessitate a high level of pumping power. Equation (2) describes the relationship between pressure drop within the system and flow, viz. resistance. Sites with high local resistance in the system contribute significantly to pressure drop, especially when small sized elements are involved (Figure 1D). All considerations so far have remained on a local scale, however, focusing on single canal system elements. In order to come up with a comprehensive functional morphological interpretation, the complete architecture of the canal system and the specific sub-elements defined in the context of hydrodynamics as functional units need to be taken into account on both the local and the organismal scale. In order to do this, two fundamental principles of resistance theory have to be considered. (1) Total resistance for serial segments is the sum of all the segments included. (2) For segments arranged in parallel, total resistance is given by the following equation.
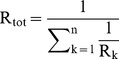
(3)As a consequence, the high resistance of numerous small sized canal segments - on any hierarchical level - turns out to make a much smaller contribution to total resistance on the organismal scale than indicated by the high individual values.

At present, the model for flow regimes in sponges [9] considers some of the physical and hydrodynamic constraints mentioned above [3], [4], [11], but with regard to morphological and architectural information is restricted to statistical morphometric data [3]. Modern imaging and analysis techniques have made detailed and even complete morphometric data available for biophysical considerations of general canal system anatomy [6], [12]–[14]. The studies in question have revealed that the architectural complexity of the canal system anatomy in leucon-type sponges is much higher than previously thought, featuring phenomena such as bypass elements or highly asymmetric branching which need to be included in an updated flow model in order to explain canal system hydrodynamics on a local scale as well as an organismal one. However, in order to obtain a sufficiently detailed picture of the hydrodynamics of the canal system to put together a new biophysical model of flow, data from a single species needs to be available for all the prerequisites mentioned above. Flow inside the canal system of sponges is influenced not only by the system's gross morphological architecture but subject too to constraints imposed by cellular elements. Most studies into sponge aquiferous systems have focused either on the architecture and morphology of the canal system in general or on the way in which choanocytes work. The present study aims to provide an overview, from a functional morphological and hydrodynamic perspective, of all relevant cellular structures within the leucon-type poriferan aquiferous system of one exemplary species.

The tropical demosponge *Tethya wilhelma* Sarà, Sarà, Nickel & Brümmer 2001 was chosen as a model on which to assess the way in which the morphology of cellular elements of the canal system relates to functional morphological aspects derived from hydrodynamic constraints. The general architecture of the canal system had already been examined for this species on an organismic scale [6], [15]. Being one of the rare sponge species continuously cultivable under laboratory conditions [16]–[19] and even exhibiting regular asexual reproduction by budding [20], *T. wilhelma* is an emerging model demosponge for various types of functional investigation including physiological, genetic and morphological studies.

Morphologically speaking, the following series of elements are considered the functional modules of the aquiferous system [21]: Ostia>(sub dermal lacunae)>incurrent canals>prosopyls>choanocyte chambers>apopyles>excurrent canals>oscule(s). Ostia are the microscopic incurrent openings into the system, while the oscule or oscules are the excurrent openings. The choanocyte chambers act as displacement pumps and generate the pressure differential which drives the water through the system [10]. Their in- and excurrent openings are called the proso- and apopyle.

There are large discrepancies in our current morphological understanding of the various elements of and cell types involved in the aquiferous system. Although some cell types (e.g. endopinacocytes and choanocytes in particular) have been studied in detail, thorough comparative cytological studies based on broad taxon sampling are scarce. The most comprehensive review is to be found in Simpson's compendium of sponge biology [21], though the information in it is unfortunately fairly general. A more recent and detailed study into cell types in demosponges focuses on systematic and evolutionary aspects of aquiferous system characters [22]. Detailed morphological studies of cell types which contribute to functionally important elements of the aquiferous system help us, when they consider the hydrodynamic environment in which such cells are found, to assess their functional role [23], [24]. This applies to apopylar cells (cone cells), central cells and any other cell type located in hydrodynamically pivotal sites in choanocyte chambers.

Theoretical and experimental investigations into choanocyte chambers have shown on the basis of choanocyte arrangement and orientation that the chambers can be understood as positive displacement pumps or, in technical terms, as peristaltic pumps [10], [11]. Experimentally and theoretically consistent models for filter feeding in sponges do exist, though definitive experimental evidence is still lacking since science currently lacks the technical observation methodologies for *in vivo* studies [1], [25]. However, in order to complement our understanding of functional morphology, the present study is intended to provide a detailed analysis of cell types within the canal system of *T. wilhelma* with respect to their impact on local flow and consequences for hydrodynamics on an organismic level.

## Results

### Canal system compartments and anatomical details

**Figure 2:**
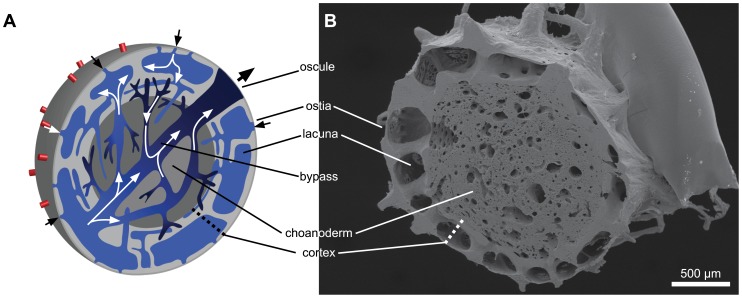
Schematic organization (A) and habitus (B) of *T. wilhelma* aquiferous system. (A) Potential flow directions in the canal system are indicated with arrows (after [15]). A color gradient from light to dark blue in the canals indicates the allocation of the corresponding elements to the incurrent and excurrent system. Due to the presence of bypasses in the canal system flow directions cannot be assigned with certainty to all sections. This might even cause backflows from the excurrent to the incurrent system. Main features/structures of the canal system are labeled in the scanning electron micrograph (B) as well as in the schematic drawing (A).

The canal system architecture in *T. wilhelma* is of the leucon type with some striking manifestations of specific canal system elements. The incurrent canal system features voluminous cortical lacunar sub-dermal cavities. This cortical lacunar network is connected to an underlying network of sub-lacunar cavities located at the choanosome/cortex boundary. Both lacunar systems consist of an extensive network of anastomosing oval-shaped/flat canals. Branching off from the lacunar- and sub-lacunar cavities, high numbers of ramifying canals lead into the choanosome. Due to the roughly globular shape of the body, the canals of the incurrent and excurrent canal systems are significantly intertwined in the choanosome region. Within the excurrent canal system the atrium region stands out by virtue of its volume and can be characterized as a larger sized canal resembling a vestibule which opens directly into the outflow opening (oscule) (Figure 2). Depending on the state of morphological (re-)organization and environmental flow conditions, varying numbers of oscules are present, from one in the majority of cases to several in more rare cases.

### Ostia

**Figure 3:**
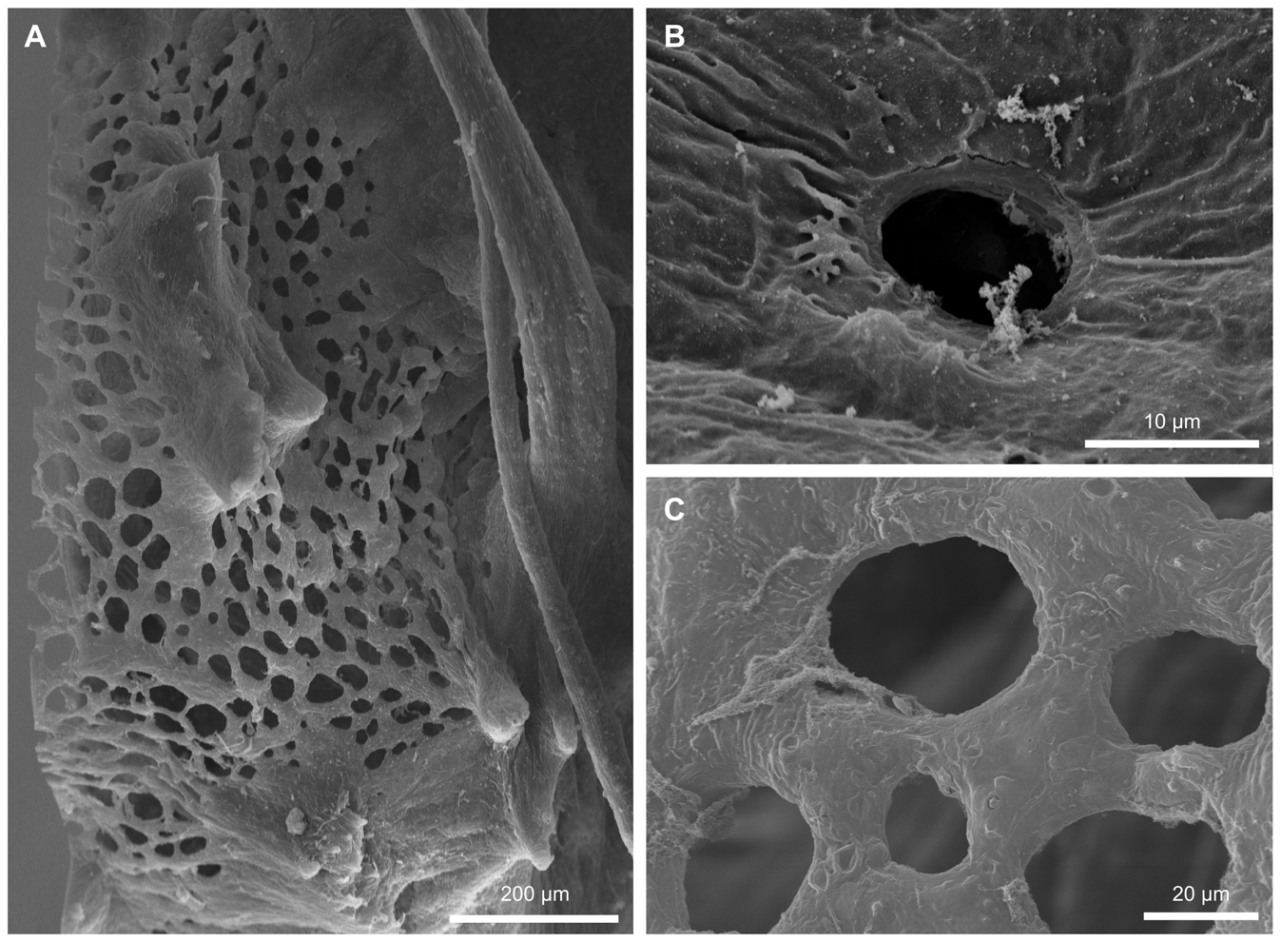
Scanning electron micrograph of an ostia pore field (A), a single ostium (B) and details of ostia in an ostia pore field (C).

Specimens of *T. wilhelma* exhibit ostia of varying sizes, with no direct correlation with body size discernible - at least not in the specimens investigated here (Figure 3). The diameters of single ostia in all the specimens studied (N = 10) ranged from fully closed to a typical maximum of <15 µm. Ostia greater than this in diameter were present only in very low numbers. Depending on environmental flow conditions, ostia appear as single openings, in small groups or as ostia fields (Figure 3A). Smaller sized ostia are formed by intracellular pores (Figure 3B, Figure S1), whereas larger ones are made up like intercellular ostia by groups of several cells (Figure 3C). In both cases the exopincocytes involved in the formation of ostia are in direct contact with adjacent exopincocytes and endopinacocytes. Where specimens of *T. wilhelma* had been cultured under steady flow conditions over a long period of time, ostia fields covering the topmost portion of the surface of the sponge were observable. In this case, ostia were generally larger (up to 43 µm). Tissue bridges between the ostia usually varied in length between 5 µm and 20 µm.

### Choanocyte chambers

**Figure 4:**
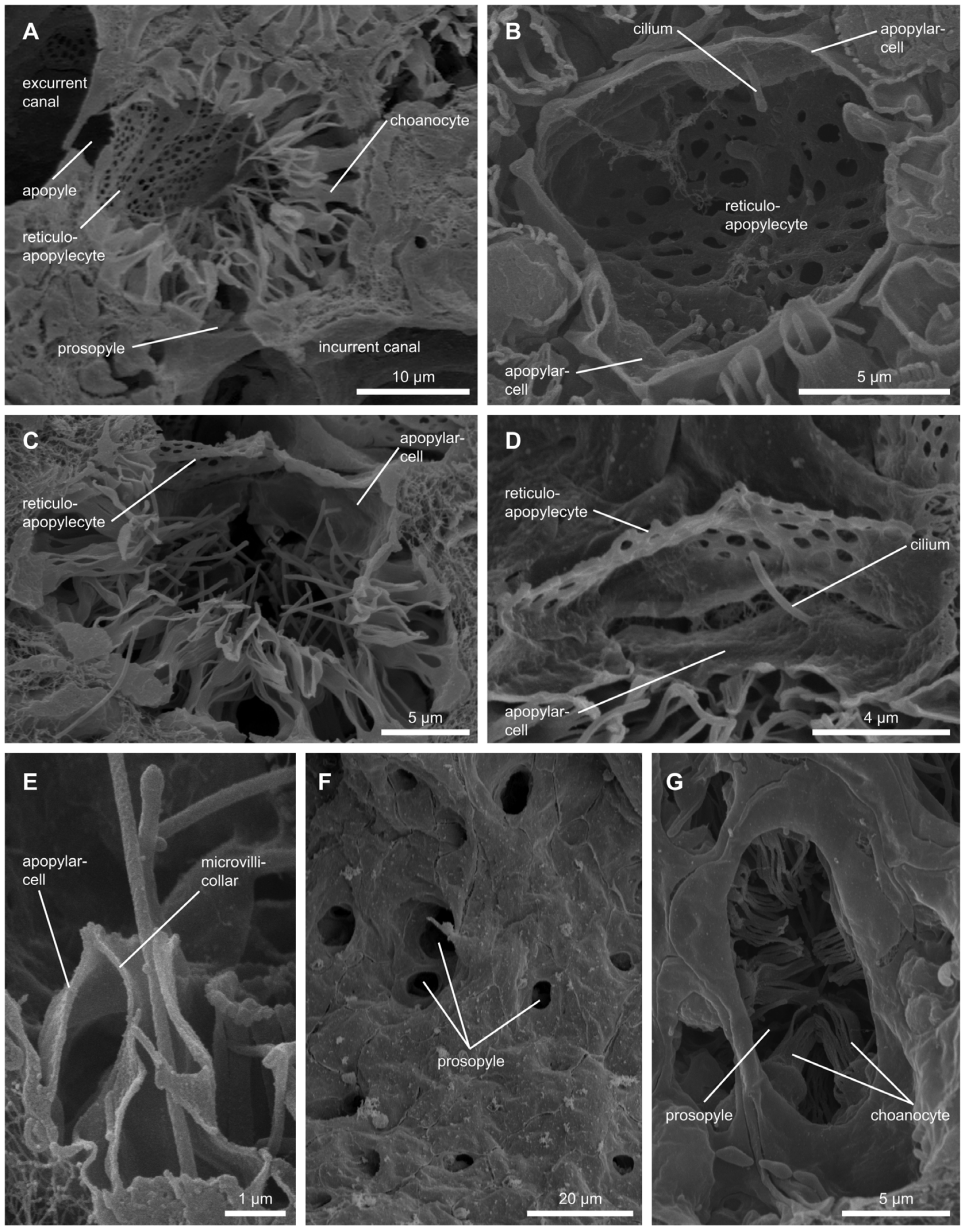
Scanning electron micrograph of cellular structures in the choanocyte chamber. (A) Overview of a choanocyte chamber connected to an incurrent- and excurrent canal with the relevant cellular prosopylar and apopylar elements and the location of the new cell type: reticuloapopylocyte. (B) Circular arrangement of apopylar cells and the position adjacent to reticuloapopylocyte. Hydrodynamic sealing of apopylar velum and microvilli collar. (C) Arrangement of cilium bearing apopylar cells, choanocytes and reticuloapopylocytes in the choanocytic apopyle. (D) Detailed view of an apopylar cell with its cilium directing into the flow at the apopyle. (E) Detailed view of the apopylar velum and microvilli collar contact side which results in a hydrodynamic sealing. (F) Overview of prosopylar openings in the incurrent canal system. (G) Pore cell forming a prosopylar opening. In the background microvilli collars of choanocytes are visible.

Choanocyte chambers are almost globular in *T. wilhelma* and possess one apopylar and one to several prosopylar openings (Figure 4A). The number of choanocytes within a choanocyte chamber is dependent on chamber size and body size (∼50–90 choanocytes/chamber, 70±13 choanocytes/chamber (N = 15 taken from 4 specimens)). The choanocytic prosopyle is formed by an interstice between adjacent choanocytes which lack filopodial extensions, which means that the prosopyle itself lacks any kind of specialized choanocytic prosopylar structure (Figure 4A).

### Prosopyles

Prosendopinacocytes form internal, single-cell pores known as pinacocytic prosopyles (Figure 4F). The mean diameter of these pore-based openings into the choanocyte chambers is about 7.4 µm. The prosendopinacocytes which form the pinacocytic prosopyle come into direct contact with the basal part of choanocyte cell bodies (Figure 4G).

### Apopyles

**Figure 5:**
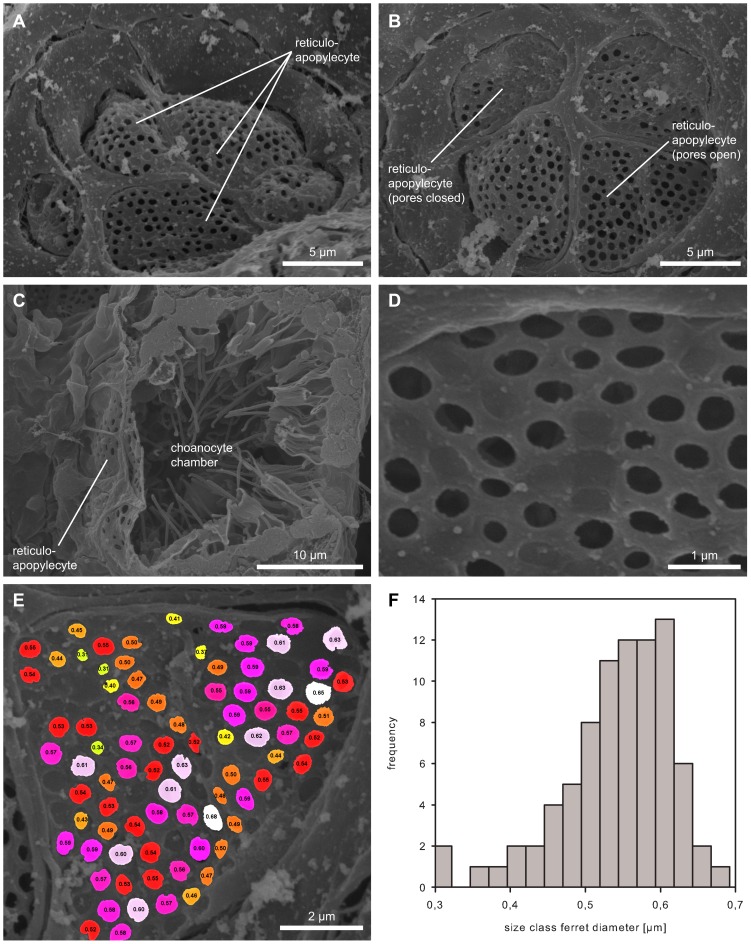
Scanning electron micrographs of reticuloapopylocytes. (A) View on reticuloapopylocytes from the excurrent canal with adjacent endopinacocytes and most of the pores open. (B) View on reticuloapopylocytes from the excurrent canal with one cell having most of the pores closed. (c) Overview of the position of reticuloapopylocytes in the apopyle (cross section through a choanocyte chamber). (D) Detailed view on pores of reticuloapopylocytes in an open and closed state. (E) Color coded and labeled ferret pore diameter of reticuloapopylocyte. (F) Distribution of ferret pore diameters in reticuloapopylocytes.

The choanocytic apopyle is formed by apopylar cells (Figure 4B–D), two to three of which (depending on the size of the choanocyte chamber) form a ring-like structure (Figure 4B). Each apopylar cell bears a single cilium 3.9 µm in length (Figure 4D). In a cross-sectional view the ring formed by apopylar cells around the apopylar opening displays a characteristic double cone shape [26] (Figure 4C). On the choanocytic face the apopylar cells come into contact with choanocytes by way of a thin velum which forms the edge of the inner part of the ring/pore structure. This velum comes into direct contact with the choanocyte microvilli collar. The single cilium of the apopylar cells projects into the apopylar opening (Figure 4B–D). Facing the apopyle the cells connect to an apopylar pore-forming apendopinacocyte, which in turn touches a hitherto undescribed cell type spanning the apopylar opening (Figure 4B, Figure 5).

### A new mesh-forming cell type within the apopyle - Reticuloapopylocyte

Reticuloapopylocytes – a previously unknown type of cell - have a high number of small intracellular pores which give them a mesh or grid-like morphology (Figure 5). These pores have openings of about 0.53 µm±0.07 µm (N = 82, taken from 1 specimen) (Figure 5E–F) and are found in an opened and closed state (Figure 5D). Reticuloapopylocytes, then, are able to adopt a gradient of opening states from totally open and highly fenestrated to partially or almost completely closed. When all reticuloapopylocyte pores are open, the functional cross-sectional area of the apopyle equals approximately 50% of the total area which would be present if the reticuloapopylocyte was absent. Typically, the cross-sectional area available to flow is much lower. The cell itself is very thin, usually below 0.5 µm, which is why the high level of fenestration leads to a grid-like morphology. Where a single reticuloapopylocyte spans the apopylar opening, it is almost circular in shape. In the case of larger apopylar openings, two or more reticuloapopylocytes form a mesh-like covering (Figure 5A–C).

Using the pore measurements presented in figure 5E–F, we calculated how reticuloapopylocytes contribute to the resistance of flow. Taking as a basis the cross-sectional area of pores and entire cells, we calculated the radius of pores and the radius of the apopylar opening. For the sake of simplification, we assumed that both were circular. By putting the measurements presented into equations 1 and 3, we calculated reticuloapopylocyte resistance to be 4.12•10^−3^ Pa s µm^−3^. In order to compare this value, we then calculated the resistance of the same apopyle opening without the reticuloapopylocyte and found it to be 3.13•10^−3^ Pa s µm^−3^. An apopylar opening with the same available cross-sectional area as the reticuloapopylocyte (12.87 µm^2^) would give rise to a single apopyle with a radius of 2.03 µm and a resistance of 5.45•10^−3^ Pa s µm^−3^. The resistance of an apopyle with a reticuloapopylocyte is therefore 1316 times greater than that of the same apopylar opening unaltered. A smaller apopyle with the same available cross-sectional area as observed in the reticuloapopylocyte would lead to a 17-fold increase in resistance compared to the reference apopyle.

### Pinacocytes

**Figure 6:**
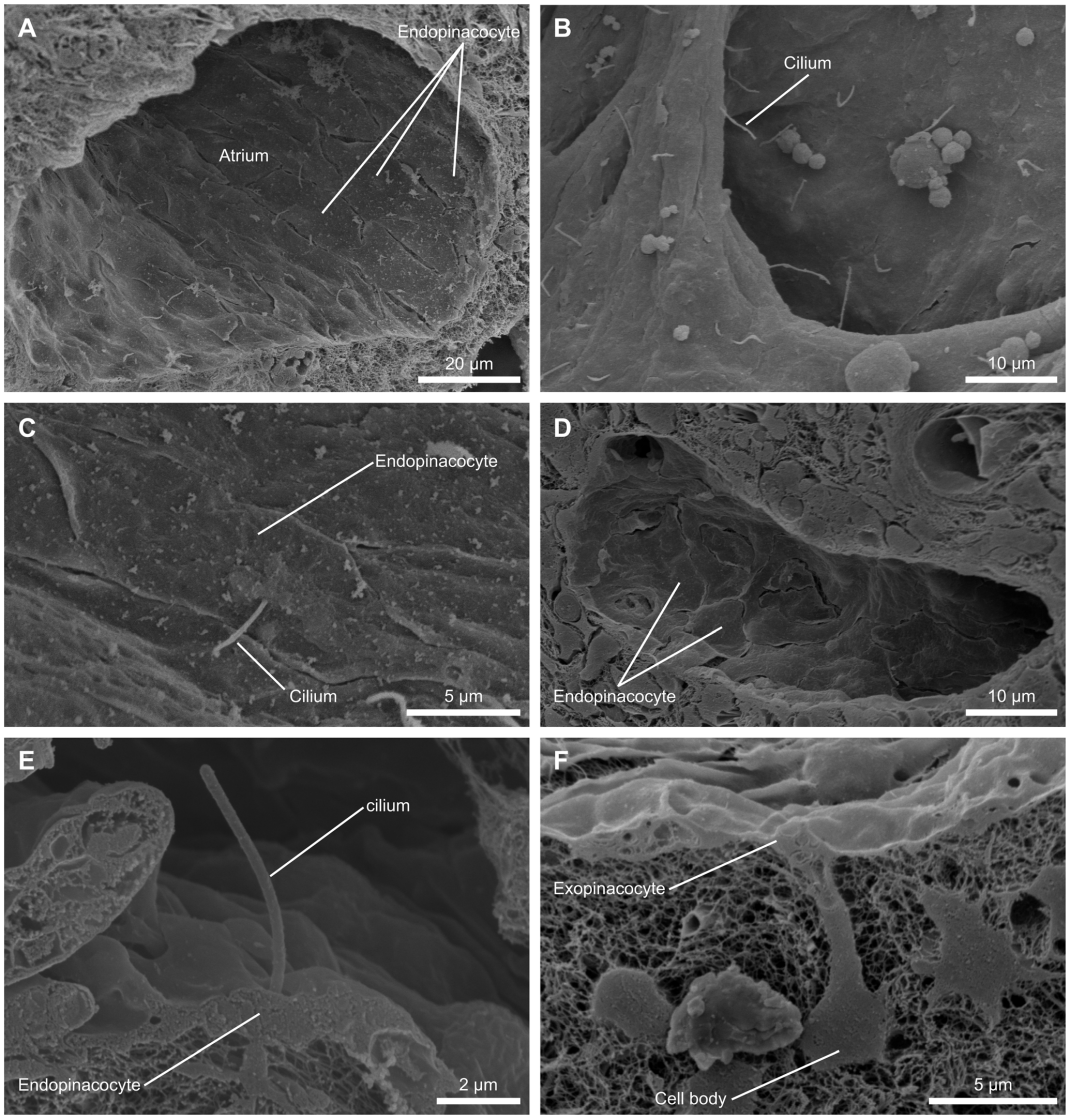
Scanning electron micrographs of pinacocytes. (A) Highly ordered apendopinacocytes in the atrium region. (B) Monociliated apendopinacocytes in the excurrent canal system. (C) Detailed view of a monociliated apendopinacocyte. (D) Prosendopinacocytes lining the walls of the incurrent canal system. (E) Detail of the cilium of an apendopinacocyte. (F) Cross section of an exopinacocyte lining the outer surface of *T. wilhelma*. Note the T-shaped umbrella like cross sectional morphology with the cell body of the pinacocyte sunk into the extra cellular matrix.

The prosendopinacocytes lining the walls of the lacunar and sublacunar cavities and the incurrent canal walls are less than 0.5 µm thick except for a small swelling incorporating the nucleus. Their overall shape is irregular and adopted to the local canal geometry (Figure 6A–D). The prosendopinacocytes in our study never displayed the T-shaped or umbrella-like morphology characteristic of exopincocytes (Figure 6F).

*T. wilhelma* possesses two types of apendopinacocytes which line the walls of excurrent canals and the atrium region, respectively. The type present in and around the atrium region bears a single 5.5 µm±0.79 µm (N = 16, taken from 4 specimens) long cilium (Figure 6C,E). Monociliated apendopinacocytes exhibit a fusiform cell morphology and appear to be arranged in a highly ordered fashion within the atrium region (Figure 6A,C). As in the case of prosendopinacocytes, the main cell body is very thin, usually below 0.5 µm, with the exception of the part holding the nucleus. Away from the atrium, monociliated apendopinacocytes become less frequent and non-ciliated apendopinacocytes start to dominate in lining the canal walls. Non-ciliated apendopinacocytes are no different on the micro morphological level to non-ciliated prosendopinacocytes.

## Discussion

### 1. Morphology

#### Ostia

The diameters displayed by ostia in *T. wilhelma* were highly variable, ranging from total closure to more than 40 µm when open. The ability to open and close ostia within a relatively short period of time for flow-regulating purposes has been documented in a number of different sponge species (e.g. [27], [28]). For this reason ostia diameters and numbers within specimens appear highly variable at any given time.

#### Pinacocytes

Biophysically, pinacocytes encounter a number of mechanical forces including shear stress and drag which are generated by flow along the canal system. Some of these forces result from direct interactions between the fluid and the pinacocyte surface which in turn contribute to general flow resistance and the resulting velocity profile. The boundary layer of the flow profile is particularly important in the context of particle feeding as it is involved in the slowdown and sedimentation of particles for phagocytosis along the canal walls [29].

The morphologies of apendopinacocytes, and most likely endopinacocytes in general, might reflect local hydrodynamics [30]. For the purposes of comparison, arterial endothelial cells have been shown under pulsatile but unidirectional laminar flow to align in the direction of flow [5]. In areas of flow separation and/or flow reversal (e.g. branching), they adopt an unaligned polygonal-shaped organization [5]. However, since our knowledge of local flow regimes in canals is very limited, it cannot yet be claimed with certainty that there is a direct correlation between endopinacocyte morphology and flow. Nevertheless, the fact that apendopinacocytes in *T. wilhelma* are aligned in an ordered way in the atrium region in particular is of great interest, for it is theoretically possible, taking fluid dynamics and morphometric data into account [6], that flow there might develop a pronounced unidirectional laminar profile.

*T. wilhelma* apendopinacocytes in and around the atrium region are monociliated. A morphologically similar cell type is characteristic of all Homoscleromorpha [22], [31], [32]. However, the monociliated endopinacocytes of Homoscleromorpha bear a much longer cilium and have been proposed to be actively involved in flow generation, something which is highly unlikely in *T. wilhelma* where the short cilium would make flow generation by apendopinacocytes relatively inefficient compared to that by choanocytes [10]. We propose as an alternative that the short apendopinacocyte cilium in *T. wilhelma* functions as a stereocilium and is involved in local flow sensing. The fact that the monociliated apendopinacocytes of the freshwater sponge *Ephydatia muelleri* (Lieberkühn, 1856), which are located in exactly the same position as in *T. wilhelma*, have recently been demonstrated to have a sensory function backs up this claim [30]. The nonmotile primary cilium in *Ephydatia muelleri* consists of 9 circularly arranged microtubule doublets (“9+0” fashion), but lacks the central ones (“9+1” fashion) characteristic of motile cilia and flagellae [30], [33].

#### Choanocyte chambers

The choanocyte chambers in *T. wilhelma* exhibit two specializations which are presumed to have a substantial impact on local and global fluid dynamics: (1) monociliated apopylar cells and (2) reticuloapopylocytes. In *T. wilhelma* apopylar cells form a ring-shaped reduction of the choanocytic apopylar opening which is double cone-shaped in cross-section. A functional morphological interpretation of the location of this cell type in a hydrodynamically pivotal site is discussed below. Apart from their role in preventing back flow, the function of apopylar cells is currently unclear, especially with regard to the cilium. However, since the cilium projects freely into the apopylar opening we propose that it is involved in flow sensing. Verifying this experimentally, however, will be technically challenging. As in the case of monociliated apendopinacocytes, ultrastructural data pertaining to microtubule arrangement might help to answer this question.

### 2. Functional Anatomy

#### Hydrodynamic situation in sections of the canal system and implications for the function of cell types

The development of ostia pore fields (see Figure 3), as observed in T. wilhelma under steady state flow conditions, can be explained as a result of fundamental fluid dynamic principles. As explained by equations 1 and 3 in the introduction, pore fields drastically reduce the total resistance of the global influx and therefore reduce global pumping energy costs. Even though the parallel arrangement of small sized elements in the canal system reduces resistance on an organismic scale, resistance in each single element remains high. Therefore, the systemic resistance of individual canal segments influences the amount of water passing through certain areas of the sponge body. This can be quantified by the term perfusion, the amount of water passing through a defined volume of the sponge body over a given time interval. Consequently, resistance is a factor which can be used directly to control the perfusion of certain areas of the sponge body and to adjust local flow. No studies to our knowledge have yet addressed this aspect of local flow regulation from a detailed theoretical and experimental perspective. However, it seems on the basis of all the available data and fluid dynamics models that a local regulation of perfusion is possible within specific areas of the sponge, and that this is most efficient in regions which (1) have a significant impact on flow resistance and (2) can be mechanically modified by the sponge. Both requirements are fulfilled when it comes to ostia and the oscule, and in principle in the case of small sized canal segments too. As T. wilhelma normally only possesses one oscule, flow theory and the continuity of flow would suggest that oscule contraction would only cause very slight variation in local flow. This is supported by studies into ostia contraction in T. wilhelma (unpublished data) and other species, which have demonstrated that single ostia can be contracted individually [34]. Unless new methodologies become available, however, it will only be possible to demonstrate this quantitatively and experimentally in a transparent sponge species which permits in situ high resolution flow measurements to be taken within the canal system. The question of whether and how small sized canal segments influence perfusion is closely related to the pronounced regular body contractions observed in T. wilhelma. Predicting the effects of canal contractions on local flow during a contraction and expansion cycle is difficult, as information on the exact dynamics of canal contractions can only be obtained indirectly from the overall kinetics inferable from time-lapse sequences [13], [35]. However, local body contractions and contraction waves across the body have been reported both for T. wilhelma and other sponges [36] and are presumed to be related to local changes in canal diameter and to result in changes in perfusion (see equation 1).

**Figure 7:**
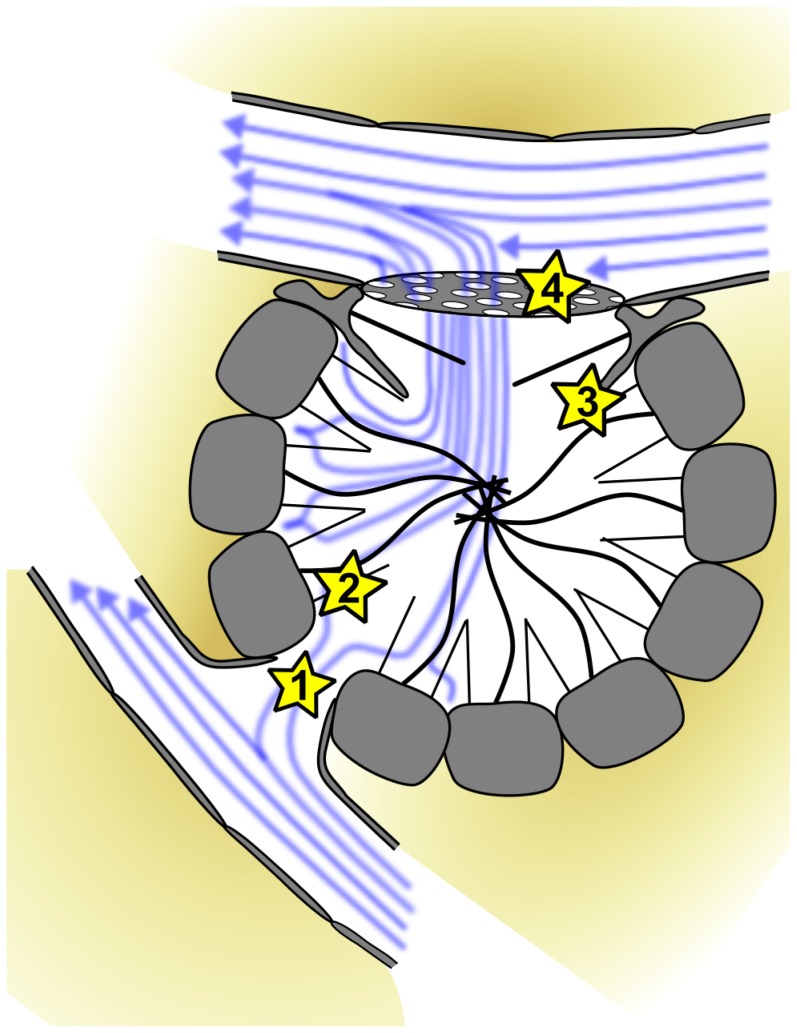
Schematic drawing of a choanocyte chamber with indicated flow directions and hydrodynamically pivotal sites (stars): 1. prosopyle, 2. microvilli collar, 3. contact side between apopylar velum of monociliated apopylar cells and microvilli collar of choanocytes at the apopylar opening, 4. reticuloapopylocyte.

In terms of local hydrodynamics, the most complex functional unit within the canal system is the choanocyte chamber. From experimental and theoretical studies into sponges and choanoflagellates, we know a good deal about particle filtering at the level of choanocytes (e.g. [2], [10], [25], [37]). However, we still lack detailed knowledge of flow fields in choanocyte chambers. A schematic drawing of simulated flow fields is given in figure 7. Hydrodynamically pivotal sites within the choanocyte chamber are marked with stars (Figure 7) and refer to structures with a significant impact on flow resistance. These include the prosopylar openings (Figure 1D), where resistance is determined by the diameter of the opening. It is presumed that the small size of these openings causes flow to accelerate compared to its velocity in adjacent canal segments. Predicting the situation for choanocytes is difficult as we lack information on how flow in the near field surrounding the choanocytes is affected by neighboring cells. In choanoflagellates, which are morphologically and functionally very similar to choanocytes microvilli collar height, density, spacing, angle and flagella length have been demonstrated to be interdependent [2]. The choanocytes in T. wilhelma have a smaller number of almost erect microvilli which are oriented parallel to each other and can be expected to reduce resistance to flow. This in turn can be expected to reduce pressure drop at the level of choanocyte chambers, if velocity is the fixed parameter or a slower flow an pumping capacity compared to choanoflagellates if pressure drop is the reference constant determining flow. Downstream in the direction of flow apopylar openings form the next anatomical structure crucial to pressure drop. In T. wilhelma, as in some other sponges [23], [26], [28], [31], [38]–[43], apopylar cells directly adjacent to the apopylar opening form a cone-shaped ring structure which makes contact with the neighboring choanocytes. The exact function of this structure is hard to pinpoint. Comparative experimental studies into flow fields around sessile and free swimming choanoflagellates might serve as a starting point. The studies in question have demonstrated that the boundary layer (e.g. the height above the substratum in a sessile choanoflagellate) has a significant influence on far and near field flow in terms of the development of eddies [16]. Applying these observations to choanocyte chambers may suggest that if no additional structures were present, eddies would develop between choanocytes and the apopylar opening. The direction of flow of eddies in this location would be opposite to the direction of outflow and would result in a significant disturbance of flow at the apopylar opening. In order to prevent the development of eddies in this location an additional boundary structure is needed. In T. wilhelma, the cone-shaped ring of apopylar cells around the opening fulfils this requirement by forming a ceiling seal with the microvilli collar tips of adjacent choanocytes, thus seeming to prevent backflow through eddies, which would significantly reduce local and global pumping efficiencies.

### 3. Functional aspects of the new cell type

From a hydrodynamic point of view, reticuloapopylocytes are the second functional morphological extravagance to be found in connection with *T. wilhelma* choanocyte chambers. Their location in the canal system and their morphology give rise to a number of hypotheses regarding their function. Reticuloapopylocytes might (1) serve as filtering devices, (2) be related to passive flow, and (3) serve as local flow-regulating devices.

A role in particle filtration, suggested by their sieve-like nature, can very likely be ruled out. We have never observed particles stuck on reticuloapopylocytes, nor witnessed any phagocytosic events. Considering the size of the pore(s) (∼0.5 µm) and the size of a typical food particle (2 µm–5 µm), we would expect the pores to be clogged by retained particles within a very short period of time. From particle feeding experiments and our understanding of hydrodynamic constraints, we know that the majority of particles are restrained with great efficiency by the microvilli collar of choanocytes at the latest [25], [44]. In other words, in terms of efficiency, an additional downstream filtering element in the form of reticuloapopylocytes is simply not necessary, which renders this potential function obsolete under parsimonious evolutionary principles.

Experimental and theoretical studies into filter feeding animals, including several sponge species, have demonstrated using a Venturi tube principle how even actively pumping species benefit from and make use of ambient flow-induced passive ventilation [11], [45], [46]. A recent work on hexactinellids provides detailed calculations of the dimensions of canal system elements (especially canal segments, choanocyte chambers and their openings) in relation to their role in fostering passive flows [46]. In this context the presence of large bypass elements [15] and the highly asymmetric nature of branching in *T. wilhelma* [6] could be interpreted as factors which promote passive flows. However, this hypothesis is speculative as the impact of bypass elements on flow patterns inside sponges is not yet well understood on either the local or the organismic scale. It is therefore currently impossible to prove or reject this hypothesis for *T. wilhelma*. What is more, a closer look at the morphology and dimensions of apopylar openings in *T. wilhelma* in the context of resistance theory does not support the hypothesis of passive ventilation by ambient flow. This is underlined by the resistance values we calculated for reticuloapopylocyte-bearing apopyles, which are about 1300 time greater than in unchanged apopyles and 8000 times greater than in the hexactinellid *Aphrocallistes vastus* [46], where ambient current-induced passive flow has been demonstrated. We would expect the much greater pressure drop/resistance generated at fenestrated apopyles in comparison to non-specialized apopylar openings to prevent the induction of passive flow through choanocyte chambers in *T. wilhelma*.

The third hypothesis regarding local flow regulation is related to the fact that individual reticuloapopylocyte intracellular pores have been observed in both an open and a closed state, and to the detection of a specific myosin-heavy chain expression pattern in this new cell type [47] which indicates its ability to actively modify its state of opening. In this it is strikingly reminiscent of intracellular ostia, which possess the ability to open and close relatively rapidly in order to regulate flow [48]–[50]. Altering the available cross-sectional area of the apopyle by entirely or partially closing individual pores changes the resistance of the apopyle. Closing pores leads to (has the capacity to lead to?) a reduction in the volume of flow and possibly even to a complete shutdown of individual choanocyte chambers in distinct areas of the sponge body. A reduction in the volume of flow at an apopyle will result in a change in the perfusion of the portion of the sponge body in question. The ability to alter flow rates on a local scale with consequences on the regional and even organismal levels qualifies the reticuloapopylocyte as a simple and highly precise fine-tuning device. Theoretically, reticuloapopylocytes permit a gradual adjustment of resistance at the apopyle by closing increasing numbers of pores to create an almost continuous decrease in flow. However, as these cells are to be found deep in the sponge body and are thus not accessible to *in vivo* light microscopy, direct experimental evidence to back up or refute this hypothesis will be difficult to obtain.

### 4. Functional constraints in the evolution of apopylar elements

Body contraction-expansion cycles have been demonstrated in representatives of all four major lineages of sponges ([36] and Nickel unpublished data). Of all the species studied so far, the amplitude and frequency of body contractions have been highest in *T. wilhelma* [13], [36]. The primary effectors of body contraction are endopinacocytes [35]. In the course of a body contraction cycle the canal lumen disappears almost entirely. The change in canal diameter leads to an increase in resistance in the canal system. This change in the hydrodynamic situation in the canal system during a body contraction cycle gives rise to three different functional constraints with regard to the evolution of apopylar elements: (1) Risk of damage to canal system elements caused by increasing pressure in the contraction phase. (2) A need to modify the perfusion of body parts, something which can be influenced by contraction and expansion phases (3) A need to generate increased Gauge pressure during the inflation of the canal system in the second kinetic phase (see [13], [35]) of the expansion cycle.

An increase in Gauge pressure within the canal system during the relatively rapid contraction phase is the result of cumulative resistance caused by the reduction in canal diameter and the presence of just a single oscule through which all residual water has to be expelled. The increased Gauge pressure leads to constraint (1), which primarily affects all delicate structures in the canal system (e.g. choanocytes). From a technical point of view the solution would be a pressure regulator. In a very simple way in *T. wilhelma*, the reticuloapopylocytes constitute just such pressure regulators. A comparable role has been demonstrated for the morphologically highly similar sieve plates in the phloem of plants [51].

The exact role of body contractions in sponges is unclear. One hypothesis proposes a physiological need to flush the canal system by exchanging all the water in the aquiferous system in the course of a body contraction cycle. Experimental studies into body contraction cycles in different sponge species have demonstrated the presence of contraction waves which travel over the sponge body ([35] and own unpublished data) Over the course of a body contraction cycle, canal diameters undergo alterations which result in changes in resistance. These changes affect perfusion rates, as formulated by constraint (2) on the principle described in section 2 above.

An analysis of body contraction kinetics in sponges has revealed four different sub-phases [35]. The contraction and expansion stages exhibit two distinct kinetic phases each. Endopinacocytes have been identified as effectors of contraction [35]. The two different kinetic phases of the *T. wilhelma* expansion cycle are thought to have two effectors. In the early and more rapid expansion phase elastic energy loaded into a distinct higher ordered sub-volume of the extracellular matrix is released [52]. This results in a partial inflation of the aquiferous system which enables the choanocyte chambers to start working again. In the second, much slower kinetic phase, we propose that Gauge pressure plays a role in fully inflating the canal system. Fulfilling this functional constraint (3) basically requires the presence of two specific components of the sponge aquiferous system - reticuloapopylocytes and bypass elements. Reticuloapopylocytes increase Gauge pressure by increasing resistance, while bypass elements form direct connections between the incurrent and excurrent canal system [6], [12], [14], [15]. Their function and impact on flow in sponges is still under debate, but hydrodynamics and resistance theory might shed light on their functional role in the context of body contraction cycles in *T. wilhelma*. The increased back pressure in the incurrent canal system generated by the presence of reticuloapopylocytes in pumping choanocyte chambers is coupled to the excurrent canal system via bypass elements. This increases Gauge pressure throughout the system, helping it to inflate.

A large number of the hypotheses and interpretations discussed above are based on theoretical considerations and fundamental physical rules, the morphology of specific cell types and the morphometric information available on the canal system. Again, experimental verification *in vivo* is not currently possible due to the lack of optical live imaging techniques for structures deep inside the sponge body. Non-destructive approaches, e.g. x-ray videography and tomography or magnetic resonance imaging, do not provide the required spatial and/or temporal resolution needed to simultaneously analyze morphology, flow and the kinetics of contraction. Furthermore, we are faced with highly complex interdependencies between the phenomena in question - e.g. pressure drop and gauge pressure being caused by bypasses and reticuloapopylocytes. A solution to this dilemma might be computational fluid dynamic modelling approaches based on exact canal system geometries obtained from biological entities. Depending on the effect to be studied, modeling approaches might enable us to reject and formulate new hypotheses, or even test the influence of specific structural elements by modifying the geometries used (e.g. including/excluding bypass elements). However, this would require detailed information on the morphology of the canal system, volume flow and temporal analysis data pertaining to the kinetics of body contractions.

## Conclusions

Reticuloapopylocytes, described here in *Tethya wilhelma*, represent a new and functionally distinct type of cell. On the basis of related functional morphological and hydrodynamic constraints, we evaluated a range of hypotheses pertaining to the function of this new cell and its effect on local and organismic flow conditions. Compared to our understanding of the functional morphology and influence on fluid dynamics of the other cell types discussed in the present study, our knowledge of the apopyle in leuconoid canal systems is patchy, especially when it comes to understanding its role in flow conditions on a local and organismic scale and its relationship to particle filtration in general. All the studies concerned with flow in sponges so far have focused mainly on the relationship between flow conditions and the architecture of the canal system in general, or concentrated on ecological aspects. However, if we break groups of cells in the aquiferous system down into functional units, the most interesting one is constituted by choanocytes and apopyle-related cells. The fact that a putative flow-regulating cell type is able to cut off every single choanocyte chamber and connected canal system elements from a highly parallelized canal system configuration raises the question of whether the apopyle is in fact a general regulative element in all sponges. Further research needs to focus on morphological changes in apopyles which reflect functional plasticity, e.g. during contraction events or pumping arrests. This will require a highly differentiated fixation scheme for functional states which will have to be characterized, analysed and understood in detail.

## Materials and Methods

### Sponge material

Individuals of *T. wilhelma* were sampled from the type location in the aquarium of the zoological-botanical garden ‘Wilhelma’ (Stuttgart). As *T. wilhelma* is not considered an endangered or protected species, no special sampling permits were required to retrieve material for scientific experiments from the aquarium section of the zoological-botanical garden. A continuous culture of sponges was maintained in a 180 l aquarium at 26°C using artificial seawater under a light/dark cycle of 12∶12 h. The sponges were fed regularly with commercial invertebrate food (Artifical Plancton, Aquakultur Genzel) [13].

### Scanning electron microscopy

Specimens of *T. wilhelma* were fixed overnight in a precooled iso-osmolar solution of 1.25% glutaraldehyde, followed by a contrasting step in iso-osmolar 1% OsO_4_ solution for 1.5 h. They were desilified in 5% hydrofluoric acid for 1 h and then embedded in styrenemethacrylate [53]. After semi-thin sectioning, we dissolved the plastic around the remaining sponge using xylene-treatment and dehydrated the samples in increasing concentrations of acetone. Specimens were critically point dried in an Emitech K850 CPD system and sputter coated in an Emitech K500 SC system. SEM images were taken on a Philips XL30ESEM instrument.

### Morphometric measurements

Morphometric measurements of reticuloapopylocytes and other cells were performed using ImageJ [54]. For the analysis of reticuloapopylocyte pore sizes pores were semi-automatically segmented using the level sets algorithm in Fiji [55]. The ferret diameters (min and max) and area of reticuloapopylocytes and all segmented pores were measured using functions in ImageJ.

## Supporting Information

Figure S1 **SEM image showing cell borders of exopinacocytes around an ostia opening in the outer surface of** ***T. wilhelma*** **.**
(PDF)
