# Supplementary material for: A New Flow-Regulating Cell Type in the Demosponge Tethya wilhelma – Functional Cellular Anatomy of a Leuconoid Canal System
Source: PLoS One. 2014 Nov 19;9(11):e113153. doi: 10.1371/journal.pone.0113153 (PMC4237394; doi:10.1371/journal.pone.0113153)

Figure S1: SEM image showing cell borders of exopinacocytes around an ostia opening in the outer surface of *T. wilhelma.*

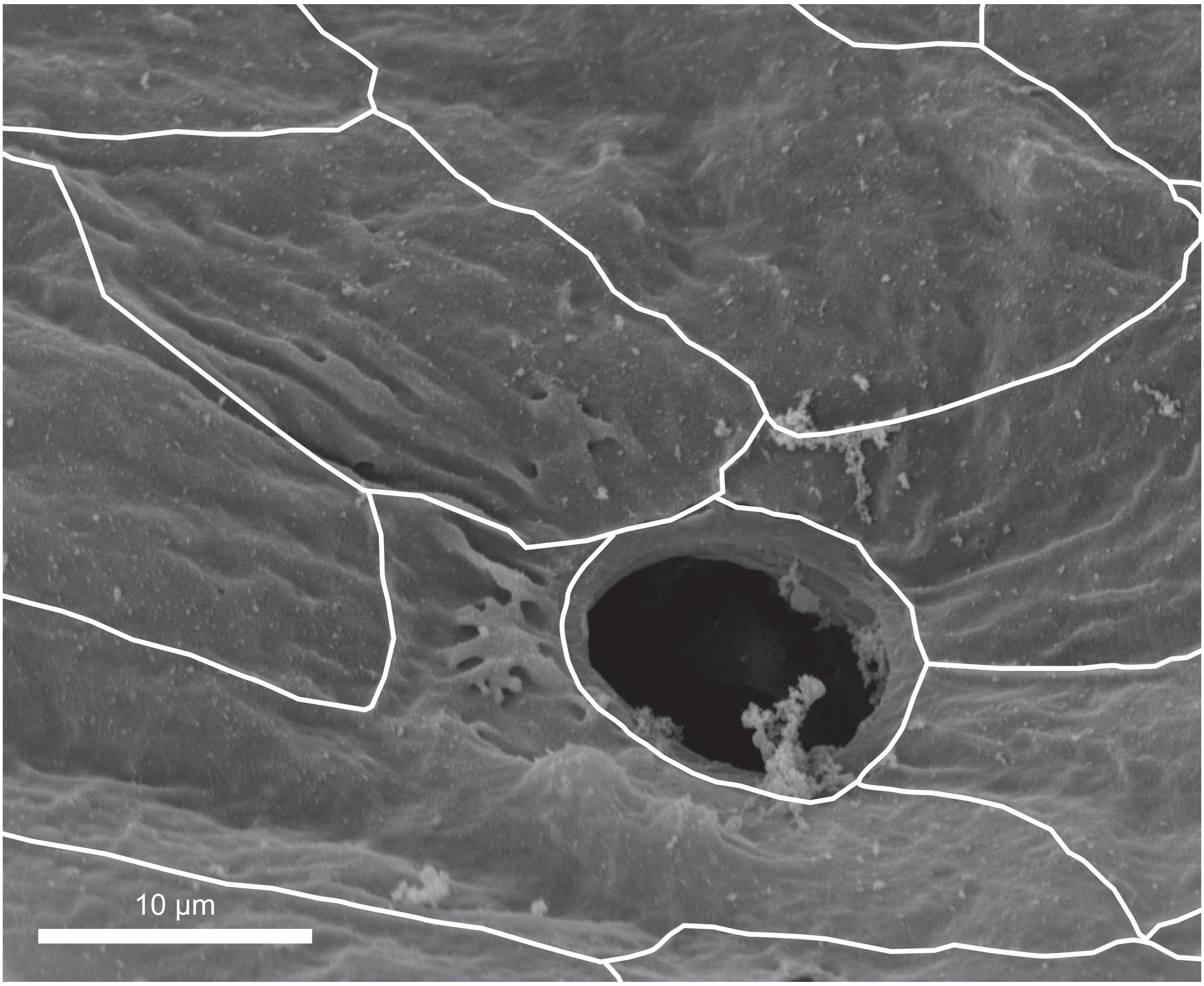

Supplement: Figure S1 — SEM image showing cell borders of exopinacocytes around an ostia opening in the outer surface of T. wilhelma . (PDF) [file pone.0113153.s001.pdf]
